# Alterations in brain structure and function associated with pediatric growth hormone deficiency: A multi-modal magnetic resonance imaging study

**DOI:** 10.3389/fnins.2022.1043857

**Published:** 2023-01-06

**Authors:** Zhibo Zhou, Yunyun Luo, Xiaoxing Gao, Yanlin Zhu, Xi Bai, Hongbo Yang, Qiuhui Bi, Shi Chen, Lian Duan, Linjie Wang, Fengying Gong, Feng Feng, Gaolang Gong, Huijuan Zhu, Hui Pan

**Affiliations:** ^1^Key Laboratory of Endocrinology of National Health Commission, State Key Laboratory of Complex Severe and Rare Diseases, Department of Endocrinology, Chinese Research Center for Behavior Medicine in Growth and Development, Peking Union Medical College Hospital, Chinese Academy of Medical Sciences and Peking Union Medical College, Beijing, China; ^2^Department of Pulmonary and Critical Care Medicine, Peking Union Medical College Hospital, Chinese Academy of Medical Sciences and Peking Union Medical College, Beijing, China; ^3^Beijing Normal University, Beijing, China; ^4^State Key Laboratory of Cognitive Neuroscience and Learning & IDG/McGovern Institute for Brain Research, Beijing Normal University, Beijing, China; ^5^Department of Radiology, Peking Union Medical College Hospital, Chinese Academy of Medical Sciences and Peking Union Medical College, Beijing, China

**Keywords:** growth hormone deficiency, insulin-like growth factor-1, intelligent quotient, diffusion tensor imaging, resting-state functional magnetic resonance imaging, structural magnetic resonance imaging

## Abstract

**Introduction:**

Pediatric growth hormone deficiency (GHD) is a disease resulting from impaired growth hormone/insulin-like growth factor-1 (IGF-1) axis but the effects of GHD on children’s cognitive function, brain structure and brain function were not yet fully illustrated.

**Methods:**

Full Wechsler Intelligence Scales for Children, structural imaging, diffusion tensor imaging, and resting-state functional magnetic resonance imaging were assessed in 11 children with GHD and 10 matched healthy controls.

**Results:**

(1) The GHD group showed moderate cognitive impairment, and a positive correlation existed between IGF-1 levels and cognitive indices. (2) Mean diffusivity was significantly increased in both corticospinal tracts in GHD group. (3) There were significant positive correlations between IGF-1 levels and volume metrics of left thalamus, left pallidum and right putamen but a negative correlation between IGF-1 levels and cortical thickness of the occipital lobe. And IGF-1 levels negatively correlated with fractional anisotropy in the superior longitudinal fasciculus and right corticospinal tract. (4) Regional homogeneity (ReHo) in the left hippocampus/parahippocampal gyrus was negatively correlated with IGF-1 levels; the amplitude of low-frequency fluctuation (ALFF) and ReHo in the paracentral lobe, postcentral gyrus and precentral gyrus were also negatively correlated with IGF-1 levels, in which region ALFF fully mediates the effect of IGF-1 on working memory index.

**Conclusion:**

Multiple subcortical, cortical structures, and regional neural activities might be influenced by serum IGF-1 levels. Thereinto, ALFF in the paracentral lobe, postcentral gyrus and precentral gyrus fully mediates the effect of IGF-1 on the working memory index.

## 1. Introduction

Growth hormone (GH) deficiency is a disease resulting from decreased secretion of GH from the anterior pituitary, with an estimated prevalence of less than 1 in 8,000 (Bao et al., 1992). The etiologies of GH deficiency (GHD) in children are versatile, and most children with isolated GHD are transient and idiopathic (Yang et al., 2017). GH can stimulate insulin-like growth factor-1 (IGF-1) secretion in liver (Ranke and Wit, 2018). The GH/IGF-1 axis plays pivotal roles in linear growth, energy homeostasis, and cognitive function (Ranke and Wit, 2018). GH receptors and IGF-1 receptors are expressed throughout human brain, including amygdala, hippocampus and parahippocampal areas, which are important in cognitive functioning (Adem et al., 1989). The important roles of GH in nervous system development and metabolism have been extensively demonstrated in the past years (Lemon and Griffiths, 2005; Waters and Blackmore, 2011; Martínez-Moreno and Arámburo, 2020; Donato et al., 2021). And IGF-1 plays critical physiological roles in reducing apoptosis, promoting synaptic plasticity and long-term proliferation of neural stem cells (Åberg, 2010; Supeno et al., 2013). As previously reported, IGF-1 signaling plays a crucial part in early brain development, neurogenesis, and brain remodeling (Wrigley et al., 2017).

The important role of GH/IGF-1 axis in normal brain morphology and function had been investigated in recent years. A previous study revealed that brain structure and function were influenced by IGF-1, and impaired IGF-1 signaling in aging people leads to significant alterations in the brain (Ashpole et al., 2015). Besides, in healthy male subjects, a higher level of IGF-1 correlates with better perceptual-motor and mental processing speed (Aleman et al., 1999). And old people with cognitive impairments have lower serum IGF-1 levels compared with those without (Landi et al., 2007). However, the effects of GHD on cognitive function, brain morphology and brain function had not been fully evaluated so far. It was reported that pediatric GHD patients exhibited significantly smaller globus pallidum, thalamic volumes, splenium of the corpus callosum and hippocampus compared with children with idiopathic short stature (Webb et al., 2012). In terms of white matter, children with GHD showed a significantly lower fractional anisotropy (FA) in corticospinal tracts and corpus callosum and a significantly higher mean diffusivity (MD) in left corticospinal tract (Webb et al., 2012). Recent studies based on resting-state functional magnetic resonance imaging (rs-fMRI) found that the GHD patients exhibited a decreased amplitude of low-frequency fluctuation (ALFF) in the left postcentral gyrus and superior parietal gyrus, an increased ALFF in angular gyrus and lingual gyrus, an increased ReHo in the left putamen and a decreased regional homogeneity (ReHo) in the right precentral gyrus, which might relate to their cognitive impairment (Zhang et al., 2021a,b). Besides, a significant decrease in functional connectivity density was observed in GHD children, especially in the left cerebellar lobules, right precentral gyrus and left postcentral gyrus (Hu et al., 2019). The effects of GHD on cerebral cortex function were also reported, including slower memory processing speed as a result of compensatory recruitment in dorsal prefrontal brain regions (Arwert et al., 2005). The above findings indicated that GHD had an impact on the brain functional activity. However, due to the great heterogeneity of GHD patients and control groups in different studies, the exact nature of the cognitive function and brain structure and function associated with GHD remains unclear.

In recent years, magnetic resonance imaging (MRI) was widely used in evaluation of brain structure variations in patients. T1-weighted structural MRI can provide volumetric analysis of subcortical and cortical gray matter using surface-based morphometry (SBM) method and voxel-based morphometry (VBM) method (Ashburner and Friston, 2000; Goto et al., 2022). Diffusion tensor imaging (DTI) is a method based on the anisotropic nature of water motion in white matter fibers, providing quantitative indices such as FA and MD (Assaf and Pasternak, 2008). The FA value, a measure of the degree of directionality of water motion, is a marker of microstructural integrity of fibers (Tae et al., 2018). The MD value, a measure of overall degree of water motion, being independent of direction, is sensitive to cellularity, edema and necrosis (Tae et al., 2018). Rs-fMRI is a noninvasive method to detect spontaneous neural activity fluctuations based on blood oxygenation level-dependent signals (Lv et al., 2018). The ReHo value is proportional to the centrality and coherence of regional brain activity (Lv et al., 2018). A higher ALFF value represents higher regional spontaneous neural activity. The fractional amplitude of low-frequency fluctuation (fAFLL) is another value with higher sensitivity and specificity to reflect regional spontaneous brain activity, which measures the power in low-frequency range divided by the total power to suppress those non-specific signal components (Zou et al., 2008).

In this study, the data of T1-weighted imaging, DTI, rs-fMRI and full Wechsler Intelligence Scales for Children were compared between children with isolated GHD and healthy children, to evaluate the alterations in brain morphology and function associated with pediatric GHD.

## 2. Subjects and methods

### 2.1. Participants

In this cross-sectional study, 11 children with GHD and 10 healthy controls matched for age, gender, and family social economic status were recruited from the Department of Endocrinology in Peking Union Medical College Hospital (PUMCH) (Wilson, 1993). The study protocol (S-K2120) was approved by the Ethics Committees of PUMCH. Written informed consent was obtained from all subjects.

In the GHD group, inclusion criteria included: (1) GHD was diagnosed according to the guideline for GHD (Cook and Rose, 2012); (2) peak value of GH in both levodopa stimulation test and insulin tolerance test <5 ng/ml; (3) there was no evidence of deficiency of other pituitary hormones; (4) no structural impairment of sellar mass was found on MRI; (5) all participants were prepubertal, namely testicular volume ≤4 ml in male and Tanner I breast in female patients. Exclusion criteria included: (1) Children with chronic liver or kidney diseases; (2) history of congenital heart diseases, cardiac insufficiency, skeletal malformations or chromosomes abnormalities; (3) history of epilepsy, neurological disorders, or psychiatric disorders.

Ten age, gender, and family social economic status (SES) matched healthy controls were enrolled, with a peak GH value >10 ng/ml in GH stimulation test or with normal height and serum IGF-1 levels >the 50th percentile of normal range according to bone age, gender and ethnicity (Li et al., 2009; Xu et al., 2010). All participants were right-handed and had no contraindication to MRI.

### 2.2. Clinical and neuropsychological assessment

Height and weight were measured in the early morning by the same anthropometer. Bone age was measured on the left hand. Serum levels of IGF1, thyroid stimulating hormone (TSH), free thyroxine (FT4), and free triiodothyronine (FT3) were tested in the department of the clinical laboratory in PUMCH by standard protocols. Serum IGF-1 levels were transformed into IGF-1 SDS based upon age and gender according to the reference intervals of serum IGF-1 levels in Chinese children (Xu et al., 2010). Cognitive function was assessed with *Full Wechsler Intelligence Scales for Children 4th edition (WSIC-IV)* by an experienced neurologist, including full-scale intelligence quotient (fs-IQ), processing speed index (PSI), working memory index (WMI), perceptual reasoning index (PRI), and verbal comprehension index (VCI). Family social economic status (SES) was assessed with *SES questionnaire* (Shi and Shen, 2017), including parents’ education levels and occupations.

### 2.3. MRI image acquisition

Data from structural imaging, DTI and rs-fMRI were collected using a 3.0 TMR scanner (GE DISCOVERY MR750) with an 8-channel phased-array head coil by the same operator at PUMCH. Lying rest was performed for 30 min before scanning. Foam padding was used to prevent the effects of head movement and external noise during scanning. They were instructed to stay still, relaxed and awake, and avoid any thoughts about intentional problems.

The following parameters were used in the collection of T1-weighted structural images: repetition time (TR) = 7.9 ms, echo time (TE) was set to the minimum value of the system, slice thickness = 1.3 mm, matrix size = 256 × 256, interlayer spacing = 0, NEX = 1, and flip angle = 90°. The following parameters were used in the collection of DTI: TR = 8,000 ms, TE was set to the minimum value of the system, matrix size = 128 × 128, slice thickness = 3 mm, interlayer spacing = 0, field of view (FOV) = 256 × 256 mm, b = 0, 1,000 s/mm^2^, the number of diffusion-gradient directions was 19, NEX = 1 and flip angle = 90^°^. The rs-fMRI data were collected with the parameters below: TR = 2,000 ms, TE = 30 ms, thickness = 4 mm, matrix size = 64 × 64, timepoint = 200, slice NEX = 1, interlayer spacing = 0 and flip angle = 90^°^.

### 2.4. Image processing

#### 2.4.1. Structural MRI

Surface-based morphometry on T1 structural MRI was performed using a pipeline of the CIVET software (version 1.1.9, Canada). Specifically, the native T1 image of each individual was linearly registered to standard MNI space, and then used the Non-parametric Non-uniform intensity Normalization (N3) algorithm to correct non-uniformity artifacts. Then the T1 images were segmented into background, cerebrospinal fluid, gray matter and white matter. The inner and outer gray matter surfaces were then extracted from each hemisphere, and the middle cortical surface was defined as the geometric center between the inner and outer cortical surfaces. The cortical surface area was defined as the area of the middle surface. Finally, a 30-mm surface-based Gaussian smoothing was applied on the cortical thickness map (Boucher et al., 2009).

Then VBM on T1 structural MRI was performed using the SPM VBM8 toolbox.^1^ Firstly, used linear and nonlinear transformations to normalize the native MR images into a standardized MNI space, meanwhile corrected for artifacts and intensity non-uniformity. Then the T1 images were segmented into background, cerebrospinal fluid, gray matter and white matter using prior tissue probability maps. The white matter and gray matter images were registered to the respective standard templates using the DARTEL toolbox. The white matter and gray matter volumes were computed for each subject. Finally, the gray matter and white matter images were smoothed using an 8 mm FWHM Gaussian kernel. Moreover, to evaluate the volume of subcortical gray matter nuclei, the subcortical structures were segmented using FSL FIRST,^2^ including bilateral hippocampus, thalamus, amygdala, accumbens, caudate, putamen, and pallidum.

#### 2.4.2. Diffusion MRI

The diffusion images were processed using PANDA, a pipeline tool for diffusion images (Cui et al., 2013). Briefly, the eddy current distortions and motion artifacts were corrected by applying an affine alignment of each DTI image to the b0 image. Then, the b-matrix was reoriented using the transformation matrix generated in eddy current correction. After preprocessing, the diffusion tensor was estimated for each voxel by solving the Stejskal and Tanner equation. The FA and MD were calculated for each voxel. The FA and MD maps of each subject were co-registered to corresponding native T1 images, and then nonlinearly warped into the standard template using the T1-to-template transformation. Finally, the FA and MD maps in the standard template space were resampled to 2 mm isotropic voxels. To minimize the noise induced by spatial normalization, FA and MD maps were smoothed using a 6 mm FWHM Gaussian kernel.

#### 2.4.3. Functional MRI

The fMRI images were processed using DPARSF (Chao-Gan and Yu-Feng, 2010), which was a MATLAB toolbox based on SPM8 and REST. Firstly, removed the first 10 time points and correct the slice timing and head motion. No subject was removed when the exclusion criteria were a maximum rotation of more than 3° or a maximum translation of more than 3 mm for the GHD group and more than 2° or 2 mm for the control group. After that, fMRI images in native space were normalized to the EPI template in MNI space and resampled to 3 mm isotropic voxels. ReHo was computed for each voxel. After smoothing with a 6 mm FWHM Gaussian kernel, the linear trend of time courses was removed. Then fALFF for each voxel was calculated. Finally, ALFF was calculated after applying temporally band-pass filtering (0.01–0.08 Hz) to the data. Remarkably, ALFF, fALFF, and ReHo of each participant were respectively standardized using their average ALFF, fALFF, and ReHo of the whole brain.

### 2.5. Statistical analysis

Firstly, for age, clinical and biochemical characteristics, SES and neuropsychological assessments, group differences were compared between two groups using the two-sample *t*-tests. The gender distribution of the two groups was tested using the chi-square test. Secondly, the group differences in subcortical volume were evaluated using a general linear model (GLM), adjusting for gender and age, and false discovery rate (FDR) method was applied for the multiple comparison correction. The correlations between subcortical volume and serum IGF1 levels were evaluated using GLM, with gender, age and whole brain volume as covariates, and the results were corrected with FDR. Thirdly, the group differences in cortical thickness and surface area were evaluated using GLM, adjusting for gender, age and groups, and random-field theory (RFT) was applied for the multiple comparison correction. The correlations between cortical thickness and surface area and serum IGF1 levels were evaluated using GLM, with gender, age and groups as covariates, and the results were corrected with RFT. Finally, group differences in cortical volume, FA, MD, ALFF, fALFF, and ReHo were accessed using GLM, adjusting for gender, age and groups, and AlphaSim correction was applied for the above multiple comparison correction. The correlations between serum IGF1 levels and these metrics (cortical volume, FA, MD, ALFF, fALFF, and ReHo) were evaluated using GLM, with gender, age and groups as covariates, and the results were corrected with AlphaSim. The significance threshold of the group differences was set to single-voxel *P* < 0.001 as well as cluster-level *P* < 0.05. The significance threshold of the correlations was set to *P* < 0.05.

The statistical analyses of surface maps were performed using MATLAB and SurfStat,^3^ and the statistical analyses of volume maps were performed using MATLAB and AFNI.^4^ Mediation analysis was performed using PROCESS.^5^

## 3. Results

### 3.1. Clinical and biochemical characteristics and neuropsychological assessment

As shown in Table 1, the GHD group (*n* = 11) and the control group (*n* = 10) were matched for age (10.36 ± 0.61 vs. 9.21 ± 0.54 years, *P* = 0.1761), gender (male ratio: 81.8 vs. 70.0%, *P* = 0.6350) and SES (14.45 ± 0.99 vs. 16.50 ± 0.78, *P* = 0.1267). No difference was found in the levels of TSH, FT3 or FT4 between two groups. IGF-1 SDS were significantly decreased in the GHD group (−2.41 ± 0.83 SDS vs. −1.26 ± 0.83 SDS, *P* = 0.005).

**TABLE 1:** Clinical and biochemical characteristics in two groups.

	GHD group (mean ± SD)	Control group (mean ± SD)	*P*-value
Number	11	10	–
Age (years)	10.36 ± 0.61	9.21 ± 0.54	0.176
Male (%)	81.8% (9/11)	70.0% (7/10)	0.635
Birth weight (kg)	3.48 ± 0.29	3.63 ± 0.60	0.583
Height (cm)	122.30 ± 11.39	127.14 ± 6.37	0.403
Height SDS	−2.57 ± 1.26	−1.77 ± 0.96	0.243
BMI SDS	0.32 ± 2.25	−0.61 ± 0.79	0.399
Peak GH level (μg/L)	All < 5	–	–
IGF-1 SDS	−2.41 ± 0.83	−1.26 ± 0.83	0.005
FT3 (ng/ml)	3.69 ± 0.16	3.81 ± 0.11	0.575
FT4 (ng/dl)	1.16 ± 0.06	1.27 ± 0.03	0.136
TSH (μIU/ml)	2.48 ± 0.67	2.29 ± 0.98	0.607
SES	14.45 ± 0.99	16.50 ± 0.78	0.1267

GHD, growth hormone deficiency; SD, standard deviation; BMI, body mass index; IGF-1, insulin-like growth factor-1; SDS, standard deviation score; FT3, free triiodothyronine; FT4, free serum thyroxine; TSH, thyroid stimulating hormone; SES, social economic status.

The scores in fs-IQ, PSI, WMI, PRI, and VCI were all significantly decreased in the GHD group (Table 2, all *P* < 0.05). Positive correlations were found between serum IGF-1 levels and cognitive indices, including fs-IQ, PSI and WMI (all *P* < 0.05).

**TABLE 2:** Neuropsychological assessment scores in two groups.

	GHD group (mean ± SD)	Control group (mean ± SD)	*P*-value
Full scale IQ	100.91 ± 5.04	129.10 ± 4.33	0.0005
Verbal comprehension index	35.82 ± 2.69	44.50 ± 2.62	0.0325
Perceptual reasoning index	30.82 ± 1.48	38.70 ± 1.08	0.0004
Working memory index	17.27 ± 1.19	23.40 ± 1.81	0.0096
Processing speed index	17.00 ± 1.09	22.50 ± 1.45	0.0064

GHD, growth hormone deficiency; SD, standard deviation; IQ, intelligence quotient.

### 3.2. Alteration of gray matter and correlation with IGF-1

#### 3.2.1. Subcortical gray matter

No difference was found in subcortical gray matter volume in any lobes and regions between two groups after adjusting for age, gender and whole brain volume. However, there were significant positive correlations between serum IGF-1 levels and volume metrics of the left thalamus (*r* = 0.626, FDR corrected *P* = 0.005), left pallidum (*r* = 0.635, FDR corrected *P* = 0.005), and right putamen (*r* = 0.51, FDR corrected *P* = 0.03), after adjusting for age, gender, and whole brain volume.

#### 3.2.2. Cortical gray matter

Surface-based morphometry revealed no difference in absolute cortical thickness, standardized cortical thickness, absolute cortical surface area, or standardized cortical surface area in any lobes and regions between two groups after adjusting for gender and age (all RFT corrected *P* > 0.05). Similarly, VBM revealed no difference in absolute or standardized cortical gray matter volume in any lobes and regions between two groups after adjusting for gender and age (all AlphaSim corrected *P* > 0.05).

**FIGURE 1:**
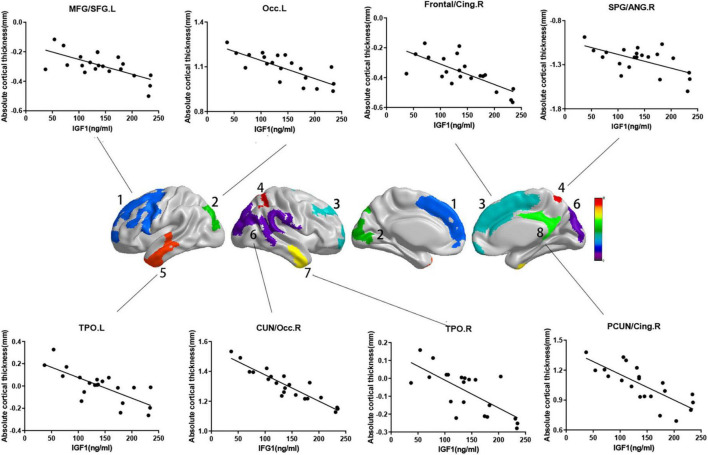
Correlations between absolute cortical thickness and serum IGF-1 levels in specific regions. Regions with *P* < 0.05 are shown. Absolute cortical thickness of above regions were negatively correlated with serum IGF-1 levels after adjusted for age, gender and group. (a) MFG/SFG.L: *r* = –0.687, *P* = 0.002; (b) Occ.L: *r* = –0.746, *P* < 0.001; (c) frontal/cing.R: *r* = –0.692, *P* = 0.001; (d) SPG/ANG.R: *r* = –0.680, *P* = 0.002; (e) TPO.L: *r* = –0.741, *P* < 0.001; (f) CUN/Occ.R: *r* = –0.864, *P* < 0.001; (g) TPO.R: *r* = –0.710, *P* = 0.001; (h) PCUN/Cing.R: *r* = –0.704, *P* = 0.001. IGF-1, insulin-like growth factor-1; MFG, middle frontal gyrus; SFG, superior frontal gyrus; Occ, occipital lobe; Frontal, frontal lobe; Cing, cingulum; SPG, superior parietal gyrus; ANG, angular gyrus; TPO, temporal lobe; CUN, cuneus; PCUN, precuneus; L, left; R, right.

**FIGURE 2:**
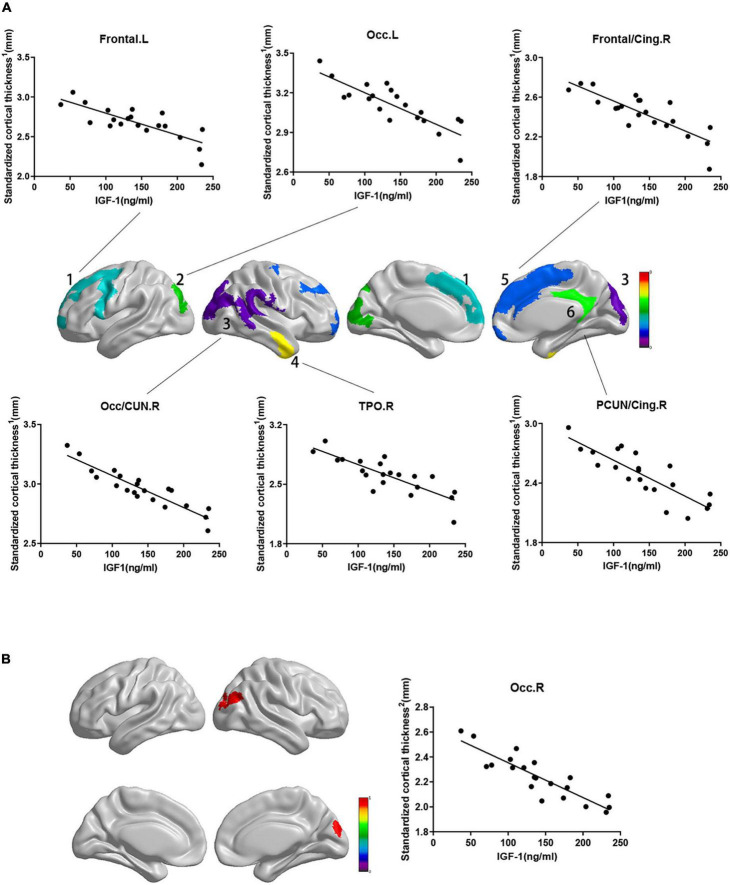
Correlations between standardized cortical thickness and serum IGF-1 levels in specific regions. Standardized cortical thickness^1^ in panel **(A)** refers to cortical thickness standardized for the whole brain volume (absolute cortical thickness divided by the whole brain volume). Standardized cortical thickness^2^ in panel **(B)** refers to cortical thickness standardized for average cortical thickness (absolute cortical thickness divided by average cortical thickness for the whole brain). Regions with *P* < 0.05 are shown. **(A)** Cortical thickness standardized for brain volume of above regions were negatively correlated with serum IGF-1 levels after adjusted for age, gender and group. (a) frontal.L: *r* = –0.693, *P* = 0.001; (b) Occ.L: *r* = –0.743, *P* < 0.001; (c) frontal/cing.R: *r* = –0.708, *P* < 0.001; (d) Occ/CUN.R: *r* = –0.870, *P* < 0.001; (e) TPO.R: *r* = –0.718, *P* = 0.001; (f) PCUN/Cing.R: *r* = –0.709, *P* = 0.001. **(B)** Cortical thickness standardized for average cortical thickness of Occ.R were negatively correlated with serum IGF-1 levels (*r* = –0.761, *P* < 0.001) after adjusted for age, gender and group. IGF-1, insulin-like growth factor-1; Frontal, frontal lobe; Occ, occipital lobe; Cing, cingulum; CUN, cuneus; TPO, temporal lobe; PCUN, precuneus; L, left; R, right.

While the cortical thickness of multiple regions was negatively correlated with serum IGF-1 levels after adjusting for gender, age and groups (Figure 1, RFT corrected *P* < 0.05). And some correlations keep stably significant after standardization for the whole brain volume (Figure 2A, RFT corrected *P* < 0.05) or average cortical thickness (Figure 2B, RFT corrected *P* < 0.05), especially in the superior occipital gyrus and middle occipital gyrus. But no significant correlation was found between serum IGF-1 levels and volume metrics of cortical gray matter, after adjusting for gender, age and groups (all AlphaSim corrected *P* > 0.05).

### 3.3. Alteration of white matter and correlation with IGF-1

**FIGURE 3:**
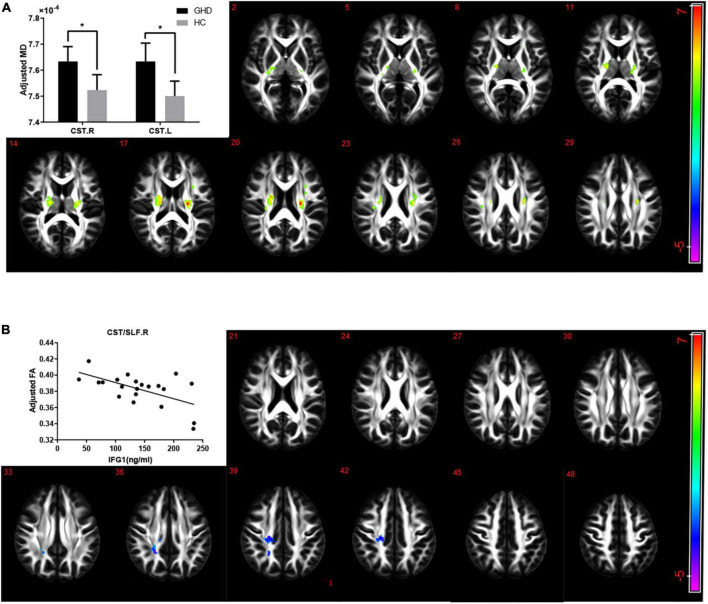
**(A)** The alterations of MD in children with GHD. The GHD group showed an increased MD in both corticospinal tracts compared with the control group after adjusting for age and gender. Hot color indicates a significant increase MD, while cold color indicates a significant decrease MD in the GHD group. **(B)** Correlations between FA and serum IGF-1 levels in CST/SLF.R. There was a significant negative correlation between serum IGF-1 levels and FA of right corticospinal tract and superior longitudinal fasciculus (*r* = –0.749, *P* < 0.001) after adjusting for age and gender. Hot color indicates a significant increase, while cold color indicates a significant decrease in those with a high serum IGF-1 levels. MD, mean diffusivity; FA, fractional anisotropy; GHD, growth hormone deficiency; IGF-1, insulin-like growth factor-1; CST, corticospinal tract; SLF, superior longitudinal fasciculus; R, right; L, left.

The GHD group showed an increased MD in both corticospinal tracts after adjusting for gender and age (Figure 3A, AlphaSim corrected *P* < 0.05). No difference was found in FA in any lobes and regions between the two groups after adjusting for gender and age. While serum IGF-1 level was negatively correlated with FA in the superior longitudinal fasciculus and right corticospinal tract after adjusting for gender and age (Figure 3B, AlphaSim corrected *P* < 0.05).

### 3.4. Alterations of resting-state brain function and correlation with IGF-1

Resting-state functional magnetic resonance imaging sequence revealed that no difference in ALFF, fALFF, or ReHo in any lobes and regions between two groups, after adjusting for gender, age and average spontaneous neural activity levels of whole brain.

**FIGURE 4:**
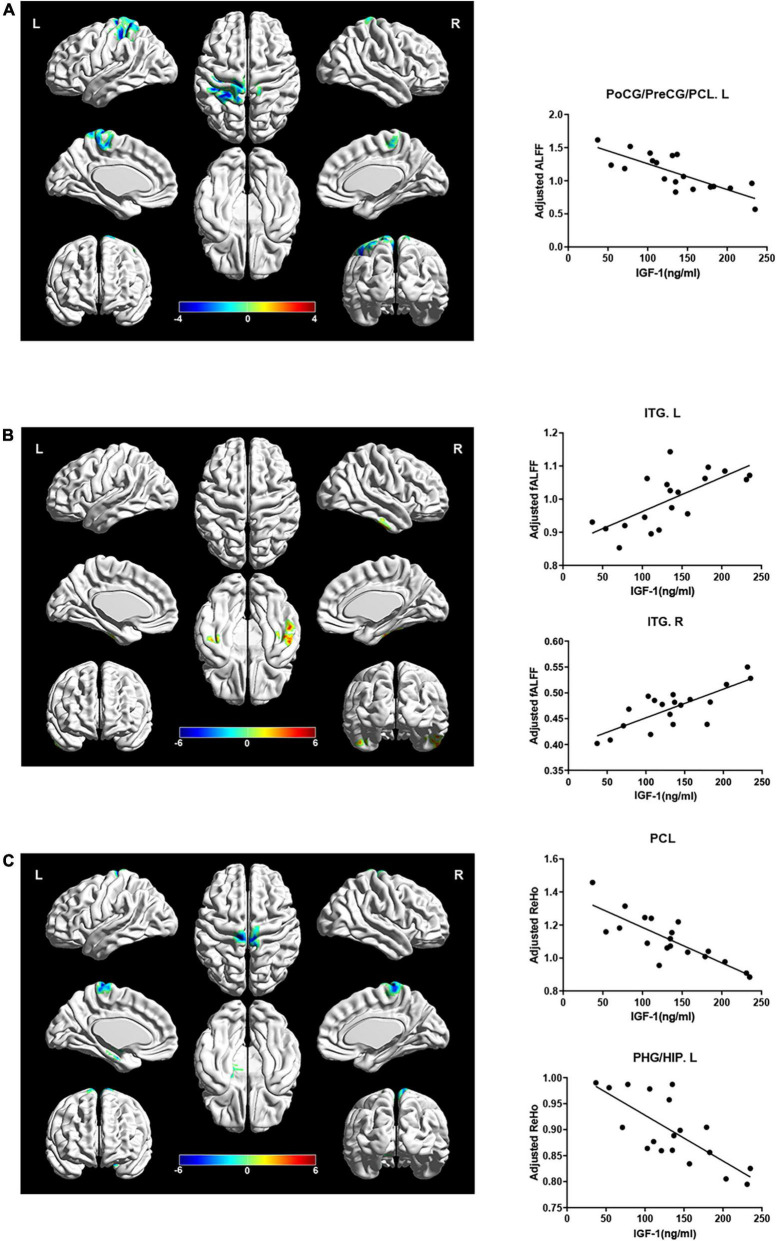
Correlations between local brain activity indexes (ALFF, fALFF, and ReHo) and serum IGF-1 levels in specific regions. Hot color indicates a significant increase, while cold color indicates a significant decrease in those with a high serum IGF-1 levels. **(A)** ALFF in PoCG/PreCG/PCL.L were negatively correlated with serum IGF-1 levels (*r* = –0.326, *P* = 0.032) after adjusted for age, gender and group. **(B)** fALFF in ITG were positively correlated with serum IGF-1 levels (ITG.L: *r* = 0.832, *P* < 0.001; ITG.R: *r* = 0.796, *P* < 0.001) after adjusted for age, gender and group. **(C)** ReHo in PCL and PHG/HIP.L were negatively correlated with serum IGF-1 levels (PCL: *r* = –0.790, *P* < 0.001; PHG/HIP.L: *r* = –0.803, *P* < 0.001) after adjusted for age, gender and group. IGF-1, insulin-like growth factor-1; ALFF, amplitude of low frequency fluctuation; PoCG, postcentral gyrus; PreCG, precentral gyrus; PCL, paracentral lobule; fALFF, fraction amplitude of low frequency fluctuation; ITG, inferior temporal gyrus; ReHo, regional homogeneity; PHG, parahippocampal gyrus; HIP, hippocampus; L, left; R, right.

However, ALFF and ReHo in the paracentral lobe, precentral gyrus and postcentral gyrus were negatively correlated with IGF-1 levels after adjusting for gender, age and groups (Figures 4A, C, AlphaSim corrected *P* < 0.05). ReHo in the left hippocampus/parahippocampal gyrus was also significantly negatively correlated with serum IGF-1 levels after adjusting for gender, age and group (Figure 4C, AlphaSim corrected *P* < 0.05). While fALFF in inferior temporal gyrus was positively correlated with IGF-1 levels after adjusting for gender, age and groups (Figure 4B, AlphaSim corrected *P* < 0.05).

### 3.5. Correlations between parameters in prior brain regions and cognitive function

Statistical analyses were performed to evaluate the correlations between parameters in prior brain regions and cognitive function. There were negative correlations between FA in the superior longitudinal fasciculus/right corticospinal tract and fs-IQ (*r* = −0.475, *P* = 0.034), VCI (*r* = −0.589, *P* = 0.006) as well as PSI (*r* = −0.454, *P* = 0.044). Besides, negative correlations were found between ALFF in both precentral gyrus/postcentral gyrus/paracentral lobe and fs-IQ (*r* = −0.474, *P* = 0.047), VCI (*r* = −0.475, *P* = 0.046) as well as WMI (*r* = −0.562, *P* = 0.015), after adjusting for age.

### 3.6. Mediation effects between IGF-1 and neuropsychological assessment scores

**FIGURE 5:**
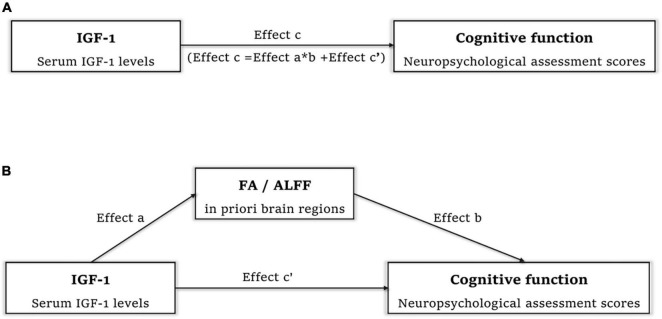
Mediation analysis to find whether FA/ALFF serve as a mediate between IGF-1 and cognitive function. **(A)** Effect c refer to total effect of IGF-1 on cognitive function. **(B)** Effect c’ refer to direct effect of IGF-1 on cognitive function. Effect a refer to effect of IGF-1 on FA in right corticospinal tract and superior longitudinal fasciculus/ALFF in both precentral gyrus, postcentral gyrus and paracentral lobe. Effect b refer to effect of FA/ALFF in above regions on cognitive function. Effect a*b refer to the effect of IGF-1 on cognitive function mediated by FA/ALFF in above regions. IGF-1, insulin-like growth factor-1; FA, fractional anisotropy; ALFF, amplitude of low frequency fluctuation.

Correlations between IGF-1 and cognitive function, IGF-1 and FA/ALFF, as well as FA/ALFF and cognitive function have been shown above. Thus, Mediation analysis was performed to find whether FA/ALFF serves as a mediate between IGF-1 and cognitive function (Figures 5A, B). And the analysis showed that ALFF in both precentral gyrus, postcentral gyrus and paracentral lobe fully mediate the effect of IGF-1 on the WMI (Table 3).

**TABLE 3:** Mediation effects between serum IGF-1 levels and neuropsychological assessment scores.

Independent variable	Mediator	Dependent variable	Effect a (*P*-value)	Effect b (*P*-value)	Effect a*b (95% CI)	Effect c’ (*P*-value)	Mediation analysis
IGF-1	FA	Full scale IQ	0.001	0.034	[−0.0505, 0.1804]	0.0647	No mediation
IGF-1	FA	Processing speed index	0.001	0.044	[−0.0187, 0.0326]	0.0402	No mediation
IGF-1	ALFF	Full scale IQ	0.031	0.047	[−0.0118, 0.2287]	0.4061	No mediation
IGF-1	ALFF	Working memory index	0.031	0.015	[0.0019, 0.0796]	0.2483	Full mediation

IGF-1, insulin-like growth factor-1; FA, fractional anisotropy; IQ, intelligence quotient; ALFF, amplitude of low frequency fluctuation.

## 4. Discussion

In this cross-sectional study, brain structure, brain function and cognitive function of GHD children had been comprehensively assessed. Our data showed that: (1) Children with GHD showed significantly decreased scores in fs-IQ, PSI, WMI, PRI, and VCI. A positive correlation existed between IGF-1 levels and cognitive indices. (2) MD significantly increased in both corticospinal tracts in children with GHD. (3) There were significantly positive correlations between IGF-1 levels and volume metrics of the left thalamus, left pallidum and right putamen, while negative correlations between IGF-1 levels and cortical thickness of multiple regions, especially in the middle and inferior occipital lobe. And serum IGF-1 levels negatively correlated with FA in the superior longitudinal fasciculus and right corticospinal tract. (4) In resting-state fMRI sequence, a significantly negative correlation was found between ReHo in the left hippocampus/parahippocampal gyrus and serum IGF-1 levels; ALFF and ReHo in the paracentral lobe, precentral gyrus and postcentral gyrus were also negatively correlated with IGF-1 levels, in which region ALFF fully mediates the effect of IGF-1 on WMI.

Gray matter structure was influenced by the GH/IGF-1 axis. We found significant positive correlations between serum IGF-1 levels and volume metrics of the left thalamus, left pallidum and right putamen, and negative correlations between IGF-1 levels and cortical thickness in multiple regions including partial areas of the occipital lobe, frontal lobe and so on, which means these specific subcortical and cortical structures were more susceptible to GH/IGF-1 axis variations. Previous studies have found a high density of GH receptors in the putamen and the thalamus (Lai et al., 1993; Creyghton et al., 2004). The putamen forms part of the dorsal striatum of basal ganglia, and plays an important role in reinforcement learning and motor control (Zhao et al., 2021). The thalamus is a structure with many important functions, including sensory and motor function, attention, memory, and emotion (Ide et al., 2015). In rats with GHD, a reduction of local cerebral glucose utilization was found in thalamic regions (Lynch et al., 2001; Sonntag et al., 2006). The brain glucose utilization was often served as an indication of neuronal activity, and the reduction of local glucose utilization might be a potential mechanism of how the impaired GH/IGF-1 axis influenced on thalamus (Webb et al., 2012). In this study, very stable correlation was found between serum IGF-1 levels and cortical thickness in the occipital lobe. The variations of cortical thickness in the partial areas of the occipital visual syncortex, parietal lobe and frontal lobe and had been reported in adults with GHD in the previous study (Yang et al., 2019). But the correlation between IGF-1 levels and cortical thickness has not been described in children with GHD before, and further research is needed on functional implications. However, we found no difference in cortical thickness or subcortical gray matter volume between two groups. For example, decreased volumes in the right hippocampus, left thalamus and right pallidum in GHD children were reported in previous study (Webb et al., 2012), while we found no such result after adjusting for age, gender and whole brain volume. Besides, some studies had reported that cortical thickness changed in different regions and lobes in adult GHD subjects (Nashiro et al., 2017; Yang et al., 2019), possibly because of different developmental stages, longer duration of GHD in adult GHD subjects or the effect of recombinant human growth hormone treatment, which means GHD may have different effects on the brain structure at different age.

The GH/IGF-1 axis also plays a significant role in white matter structure. The corticospinal tracts are of vital importance in the control of afferent inputs, spinal reflexes and motor neuron activity, which controls the precise aspects of voluntary motor function in human (Lemon and Griffiths, 2005). In this study, GHD group exhibited an increased MD in both corticospinal tracts compared with the controls, and the increased MD in the left corticospinal tract is consistent with previous reports (Webb et al., 2012). Besides, we found that serum IGF-1 levels negatively correlated with FA in the superior longitudinal fasciculus and right corticospinal tract. Increased MD or decreased FA always reflect axonal damage, demyelination or loss of white matter coherence (Shukla et al., 2011). This difference in MD between groups may be related to low serum IGF-1 levels in GHD patients. In mice, IGF-1 is important in corticospinal tract development through IGF-1 receptors and downstream signaling pathways, and corticospinal motor neurons axon outgrowth will be impaired in consequence of IGF-1 signaling interruptions (Ozdinler and Macklis, 2006). Another study reported that IGF-1 gene delivery can promote corticospinal neuronal survival in mice after central nervous system injury (Hollis et al., 2009). And it’s previously reported that children with GHD had decreased FA in the left corticospinal tract (Webb et al., 2012). Our findings show more evidences to support that impaired GH/IGF-1 axis affects corticospinal tracts development in humans, which may be associated with impaired motor skills performance in children with GHD. However how the GHD process influences the structural integrity of corticospinal tracts still needs to investigate further.

Resting-state functional magnetic resonance imaging reflects spontaneous neural activity fluctuations in human brain, and ALFF, fALFF, and ReHo show high temporal stability and test-retest reliability (Lv et al., 2018). ALFF, fALFF, and ReHo in this study had been respectively standardized for average ALFF, fALFF, and ReHo of the whole brain. We found that ALFF and ReHo in the paracentral lobe, precentral gyrus and postcentral gyrus were also negatively correlated with IGF-1 levels. While previous studies reported that GHD children had a decreased ReHo in the right precentral gyrus and a decreased ALFF in the left postcentral gyrus, but they did not perform the correlation analysis between ALFF/ReHo and serum IGF-1 levels, and they also did not adjust participants’ age and gender in these studies (Zhang et al., 2021a,b). Besides, we also found that ReHo in the left hippocampus and parahippocampal gyrus were negatively correlated with serum IGF-1 levels. There is a high density of GH receptors and IGF-1 receptors in hippocampus and parahippocampal areas (Adem et al., 1989). Being a component of the limbic system, the hippocampus plays a key role in memory, learning, emotion and spatial orientation (Bast et al., 2017). Additionally, fALFF in the inferior temporal gyrus was positively correlated with IGF-1 levels, indicating decreased regional spontaneous neural activities occurred in those with lower IGF-1 levels. The inferior temporal gyrus is a crucial structure involved in higher cognitive functions, including visual recognition, language comprehensions and emotion regulation (Lin et al., 2020). The abnormal activities in these brain areas may reflect the neurophysiological basis of cognitive deficit in children with GHD. In brief, our study supported that those with higher serum IGF-1 levels have a decreased regional spontaneous neural activity and a decreased centrality and coherence of regional brain activity in specific gyri and lobes in the resting state. Hence, IGF-1 signaling is believed to be important in regional spontaneous neural activity in these specific areas in resting state.

Children with GHD showed significantly decreased scores in fs-IQ, PSI, WMI, PRI and VCI. And serum IGF-1 levels were positively correlated with the following cognitive indices: fs-IQ, PSI, and WMI. The variations of fs-IQ, PSI, and WMI were consistent with previous study (Webb et al., 2012) since GH/IGF-1 axis was believed to be important in cognitive function. However, how the GHD caused these cognitive impairments is still unknown. Up to now, this is the first study to evaluate cognitive function, brain structure and function in the same GHD children cohort, simultaneously using neuropsychological assessment, T1W, DTI, and resting-state fMRI, for which we found the correlations between IGF-1 and cognitive function, IGF-1 and brain structure/function, as well as brain structure/function and cognitive function. Thus, we performed the mediation analysis to investigate whether brain structure or function mediates the effect of IGF-1 on cognitive function. We found a very important result that ALFF in the paracentral lobe, postcentral gyrus and precentral gyrus fully mediates the effect of IGF-1 on the working memory index. The paracentral lobe, postcentral gyrus and precentral gyrus compose the central lobe, corresponding to the sensorimotor cortex, is one of the most important areas of the brain (Chauhan et al., 2021). More and more evidences proved that primary motor cortex also plays an important role in higher cognitive processes besides motor control (Bhattacharjee et al., 2021). Additionally, in rats with cognitive deficits caused by traumatic brain injury, IGF-1 gene therapy can reduce oxidative stress markers levels in motor cortex and restore their working memory performance to similar values regarding control, which indicates IGF-1 have important effects on motor cortex and working memory (Montivero et al., 2021). The increased neural activity of central lobe in GHD children observed in our study may indicate increased strategic or attentional recruitment of these brain areas, which may be a compensatory response to decreased working memory. Previous study reported impaired memory performance in GHD patients which greatly reducing their quality of life (Szarka et al., 2021). Our study provided a novel possible mechanism for explaining why those GHD children have impaired working memory, but the more specific causal inference still needs to be further explored.

This study has several limitations. First one is the small number of subjects. GHD is relatively rare with an incidence of <1/8,000. And we used more rigorous inclusion criteria in diagnosis (GH peak < 5 ng/ml) and age range compared with previous studies (Webb et al., 2012; Zhang et al., 2021a,b). These caused difficulties in recruitment but kept a good homogeneity in the etiology, age, stages of brain development and prepubertal stages, providing a general uniform background for brain structure, brain function and cognitive function evaluation. Secondly, cognitive function was only evaluated with *WSIC-IV* in this study, thus the behavior problems, executive function and social function were not assessed, for which more cognitive assessment measures can be performed in a future study. Thirdly, this is a cross-sectional study and provided no information on causality between GHD and changes in cognitive, brain structure and brain function, which needs further investigations in the future.

In summary, moderate cognitive impairment in fs-IQ, VCI, WMI, PRI, and PSI were shown in GHD children, suggesting a possible protective effect of the GH/IGF-1 axis in cognitive function. Multiple subcortical and cortical structures and regional brain activities might be under the influence of serum IGF-1 levels. And more interestingly, ALFF in both the precentral gyrus, postcentral gyrus and paracentral lobe fully mediates the effect of IGF-1 on the WMI. These findings provide novel insights into potential targets of the GH/IGF-1 axis in the central nervous system and more evidence of IGF-1 being important to the development of brain structure and function in children.

## Data availability statement

The raw data supporting the conclusions of this article will be made available by the authors, without undue reservation.

## Ethics statement

The studies involving human participants were reviewed and approved by the Institutional Review Board (IRB) of Peking Union Medical College Hospital (PUMCH). Written informed consent to participate in this study was provided by the participants’ legal guardian/next of kin.

## Author contributions

ZZ and YL analyzed the MRI image data and drafted the manuscript. XG, YZ, and XB collected and collated the clinical information and MRI image data. HY, SC, LD, and LW were responsible for recruitment of patients into group. FG was responsible for clinical and biochemical data analysis. FF helped with the MRI image acquisition. QB and GG helped with image data analysis and designed image data collection process. HZ and HP designed the study protocol and revised this manuscript. All authors contributed to the article and approved the submitted version.

## Footnotes

1
https://www.fil.ion.ucl.ac.uk/spm/

2
https://fsl.fmrib.ox.ac.uk/fsl/fslwiki/FIRST

3
http://www.math.mcgill.ca/keith/surfstat/

4
http://afni.nimh.nih.gov/afni/

5
www.processmacro.org

## References

[B1] Åberg D. (2010). Role of the growth hormone/insulin-like growth factor 1 axis in neurogenesis. *Endocr. Dev.* 17 63–76. 10.1159/000262529 19955757

[B2] Adem A. Jossan S. S. D’argy R. Gillberg P. G. Nordberg A. Winblad B. (1989). Insulin-like growth factor 1 (Igf-1) receptors in the human brain: Quantitative autoradiographic localization. *Brain Res.* 503 299–303. 10.1016/0006-8993(89)91678-8 2557967

[B3] Aleman A. Verhaar H. J. De Haan E. H. De Vries W. R. Samson M. M. Drent M. L. (1999). Insulin-like growth factor-I and cognitive function in healthy older men. *J. Clin. Endocrinol. Metab.* 84 471–475. 10.1210/jcem.84.2.5455 10022403

[B4] Arwert L. I. Veltman D. J. Deijen J. B. Van Dam P. S. Delemarre-Van Dewaal H. A. Drent M. L. (2005). Growth hormone deficiency and memory functioning in adults visualized by functional magnetic resonance imaging. *Neuroendocrinology* 82 32–40. 10.1159/000090123 16330884

[B5] Ashburner J. Friston K. J. (2000). Voxel-based morphometry–the methods. *Neuroimage* 11 805–821. 10.1006/nimg.2000.0582 10860804

[B6] Ashpole N. M. Sanders J. E. Hodges E. L. Yan H. Sonntag W. E. (2015). Growth hormone, insulin-like growth factor-1 and the aging brain. *Exp. Gerontol.* 68 76–81. 10.1016/j.exger.2014.10.002 25300732 PMC4388761

[B7] Assaf Y. Pasternak O. (2008). Diffusion tensor imaging (Dti)-based white matter mapping in brain research: A review. *J. Mol. Neurosci.* 34 51–61. 10.1007/s12031-007-0029-0 18157658

[B8] Bao X. L. Shi Y. F. Du Y. C. Liu R. Deng J. Y. Gao S. M. (1992). Prevalence of growth hormone deficiency of children in Beijing. *Chin. Med. J. (Engl)* 105 401–405. 1499371

[B9] Bast T. Pezze M. Mcgarrity S. (2017). Cognitive deficits caused by prefrontal cortical and hippocampal neural disinhibition. *Br. J. Pharmacol.* 174 3211–3225. 10.1111/bph.13850 28477384 PMC5595754

[B10] Bhattacharjee S. Kashyap R. Abualait T. Annabel Chen S. H. Yoo W. K. Bashir S. (2021). The role of primary motor cortex: More than movement execution. *J. Mot. Behav.* 53 258–274. 10.1080/00222895.2020.1738992 32194004

[B11] Boucher M. Whitesides S. Evans A. (2009). Depth potential function for folding pattern representation, registration and analysis. *Med. Image Anal.* 13 203–214. 10.1016/j.media.2008.09.001 18996043

[B12] Chao-Gan Y. Yu-Feng Z. (2010). Dparsf: A matlab toolbox for “Pipeline”. Data analysis of resting-state fmri. *Front. Syst. Neurosci.* 4:13. 10.3389/fnsys.2010.00013 20577591 PMC2889691

[B13] Chauhan P. Rathawa A. Jethwa K. Mehra S. (2021). “The anatomy of the cerebral cortex,” in *Cerebral Ischemia*, ed. Pluta R. (Brisbane, QL: Exon Publications). 10.36255/exonpublications.cerebralischemia.2021.cerebralcortex 34905314

[B14] Cook D. M. Rose S. R. (2012). A review of guidelines for use of growth hormone in pediatric and transition patients. *Pituitary* 15 301–310. 10.1007/s11102-011-0372-6 22271255

[B15] Creyghton W. M. Van Dam P. S. Koppeschaar H. P. (2004). The role of the somatotropic system in cognition and other cerebral functions. *Semin. Vasc. Med.* 4 167–172. 10.1055/s-2004-835375 15478038

[B16] Cui Z. Zhong S. Xu P. He Y. Gong G. (2013). Panda: A pipeline toolbox for analyzing brain diffusion images. *Front. Hum. Neurosci.* 7:42. 10.3389/fnhum.2013.00042 23439846 PMC3578208

[B17] Donato J. Jr. Wasinski F. Furigo I. C. Metzger M. Frazão R. (2021). Central regulation of metabolism by growth hormone. *Cells* 10:129. 10.3390/cells10010129 33440789 PMC7827386

[B18] Goto M. Abe O. Hagiwara A. Fujita S. Kamagata K. Hori M. (2022). Advantages of using both voxel- and surface-based morphometry in cortical morphology analysis: A review of various applications. *Magn. Reson. Med. Sci.* 21 41–57. 10.2463/mrms.rev.2021-0096 35185061 PMC9199978

[B19] Hollis E. R. II Lu P. Blesch A. Tuszynski M. H. (2009). Igf-I gene delivery promotes corticospinal neuronal survival but not regeneration after adult Cns injury. *Exp. Neurol.* 215 53–59. 10.1016/j.expneurol.2008.09.014 18938163 PMC2632606

[B20] Hu Y. Liu X. Chen X. Chen T. Ye P. Jiang L. (2019). Differences in the functional connectivity density of the brain between individuals with growth hormone deficiency and idiopathic short stature. *Psychoneuroendocrinology* 103 67–75. 10.1016/j.psyneuen.2018.12.229 30658340

[B21] Ide S. Kakeda S. Korogi Y. (2015). [Anatomy of the thalamus]. *Brain Nerve* 67 1459–1469. 26618760 10.11477/mf.1416200323

[B22] Lai Z. Roos P. Zhai O. Olsson Y. Fhölenhag K. Larsson C. (1993). Age-related reduction of human growth hormone-binding sites in the human brain. *Brain Res.* 621 260–266. 10.1016/0006-8993(93)90114-3 8242339

[B23] Landi F. Capoluongo E. Russo A. Onder G. Cesari M. Lulli P. (2007). Free insulin-like growth factor-I and cognitive function in older persons living in community. *Growth Horm. IGF Res.* 17 58–66. 10.1016/j.ghir.2006.11.001 17208483

[B24] Lemon R. N. Griffiths J. (2005). Comparing the function of the corticospinal system in different species: Organizational differences for motor specialization? *Muscle Nerve* 32 261–279. 10.1002/mus.20333 15806550

[B25] Li H. Ji C. Y. Zong X. N. Zhang Y. Q. (2009). [Height and weight standardized growth charts for Chinese children and adolescents aged 0 to 18 years]. *Zhonghua Er Ke Za Zhi* 47 487–492. 19951507

[B26] Lin Y. H. Young I. M. Conner A. K. Glenn C. A. Chakraborty A. R. Nix C. E. (2020). Anatomy and white matter connections of the inferior temporal gyrus. *World Neurosurg.* 143 e656–e666. 10.1016/j.wneu.2020.08.058 32798785

[B27] Lv H. Wang Z. Tong E. Williams L. M. Zaharchuk G. Zeineh M. (2018). Resting-state functional mri: Everything that nonexperts have always wanted to know. *Ajnr. Am. J. Neuroradiol.* 39 1390–1399. 10.3174/ajnr.A5527 29348136 PMC6051935

[B28] Lynch C. D. Lyons D. Khan A. Bennett S. A. Sonntag W. E. (2001). Insulin-like growth factor-1 selectively increases glucose utilization in brains of aged animals. *Endocrinology* 142 506–509. 10.1210/endo.142.1.8053 11145617

[B29] Martínez-Moreno C. G. Arámburo C. (2020). Growth hormone (Gh) and synaptogenesis. *Vitam Horm* 114 91–123. 10.1016/bs.vh.2020.04.001 32723552

[B30] Montivero A. J. Ghersi M. S. Silvero C. M. Artur De La Villarmois E. Catalan-Figueroa J. Herrera M. (2021). Early Igf-1 Gene therapy prevented oxidative stress and cognitive deficits induced by traumatic brain injury. *Front. Pharmacol.* 12:672392. 10.3389/fphar.2021.672392 34234671 PMC8255687

[B31] Nashiro K. Guevara-Aguirre J. Braskie M. N. Hafzalla G. W. Velasco R. Balasubramanian P. (2017). Brain structure and function associated with younger adults in growth hormone receptor-deficient humans. *J. Neurosci.* 37 1696–1707. 10.1523/JNEUROSCI.1929-16.2016 28073935 PMC5320603

[B32] Ozdinler P. H. Macklis J. D. (2006). Igf-I specifically enhances axon outgrowth of corticospinal motor neurons. *Nat. Neurosci.* 9 1371–1381. 10.1038/nn1789 17057708

[B33] Ranke M. B. Wit J. M. (2018). Growth hormone - past, present and future. *Nat. Rev Endocrinol.* 14 285–300. 10.1038/nrendo.2018.22 29546874

[B34] Shi B. Shen J. (2017). The relationships among family Ses, intelligence, intrinsic motivation and creativity. *Psychol. Dev. Educ.* 23 30–34.

[B35] Shukla D. K. Keehn B. Müller R. A. (2011). Tract-specific analyses of diffusion tensor imaging show widespread white matter compromise in autism spectrum disorder. *J. Child Psychol. Psychiatry* 52 286–295. 10.1111/j.1469-7610.2010.02342.x 21073464 PMC4547854

[B36] Sonntag W. E. Bennett C. Ingram R. Donahue A. Ingraham J. Chen H. (2006). Growth hormone and Igf-I modulate local cerebral glucose utilization and Atp levels in a model of adult-onset growth hormone deficiency. *Am. J. Physiol. Endocrinol. Metab.* 291 E604–E610. 10.1152/ajpendo.00012.2006 16912061

[B37] Supeno N. E. Pati S. Hadi R. A. Ghani A. R. Mustafa Z. Abdullah J. M. (2013). Igf-1 acts as controlling switch for long-term proliferation and maintenance of Egf/Fgf-responsive striatal neural stem cells. *Int. J. Med. Sci.* 10 522–531. 10.7150/ijms.5325 23532711 PMC3607237

[B38] Szarka N. Szellar D. Kiss S. Farkas N. Szakacs Z. Czigler A. (2021). Effect of growth hormone on neuropsychological outcomes and quality of life of patients with traumatic brain injury: A systematic review. *J. Neurotrauma* 38 1467–1483. 10.1089/neu.2020.7265 33677992 PMC8672110

[B39] Tae W. S. Ham B. J. Pyun S. B. Kang S. H. Kim B. J. (2018). Current clinical applications of diffusion-tensor imaging in neurological disorders. *J. Clin. Neurol.* 14 129–140. 10.3988/jcn.2018.14.2.129 29504292 PMC5897194

[B40] Waters M. J. Blackmore D. G. (2011). Growth hormone (Gh), brain development and neural stem cells. *Pediatr. Endocrinol. Rev.* 9 549–553. 22397139

[B41] Webb E. A. O’reilly M. A. Clayden J. D. Seunarine K. K. Chong W. K. Dale N. (2012). Effect of growth hormone deficiency on brain structure, motor function and cognition. *Brain* 135 216–227. 10.1093/brain/awr305 22120144

[B42] Wilson J. D. (1993). Peking union medical college hospital, a palace of endocrine treasures. *J. Clin. Endocrinol. Metab.* 76 815–816. 10.1210/jc.76.4.815 8473387

[B43] Wrigley S. Arafa D. Tropea D. (2017). Insulin-like growth factor 1: At the crossroads of brain development and aging. *Front. Cell Neurosci.* 11:14. 10.3389/fncel.2017.00014 28203146 PMC5285390

[B44] Xu S. Gu X. Pan H. Zhu H. Gong F. Li Y. (2010). Reference ranges for serum Igf-1 and Igfbp-3 levels in Chinese children during childhood and adolescence. *Endocr. J.* 57 221–228. 10.1507/endocrj.K09E-200 20051649

[B45] Yang H. Li K. Liang X. Gu B. Wang L. Gong G. (2019). Alterations in cortical thickness in young male patients with childhood-onset adult growth hormone deficiency: A morphometric MRI study. *Front. Neurosci.* 13:1134. 10.3389/fnins.2019.01134 31695595 PMC6817473

[B46] Yang H. Zhu H. Yan K. Pan H. (2017). Childhood-onset adult growth hormone deficiency: Clinical, hormonal, and radiological assessment in a single center in China. *Horm. Res. Paediatr.* 88 155–159. 10.1159/000478527 28719905

[B47] Zhang F. Hua B. Wang M. Wang T. Ding Z. Ding J. R. (2021a). Regional homogeneity abnormalities of resting state brain activities in children with growth hormone deficiency. *Sci. Rep.* 11:334. 10.1038/s41598-020-79475-9 33432029 PMC7801452

[B48] Zhang F. Hua B. Wang T. Wang M. Ding Z. X. Ding J. R. (2021b). Abnormal amplitude of spontaneous low-frequency fluctuation in children with growth hormone deficiency: A resting-state functional magnetic resonance imaging study. *Neurosci. Lett.* 742:135546. 10.1016/j.neulet.2020.135546 33290838

[B49] Zhao Z. Yao S. Zweerings J. Zhou X. Zhou F. Kendrick K. M. (2021). Putamen volume predicts real-time fmri neurofeedback learning success across paradigms and neurofeedback target regions. *Hum. Brain Mapp.* 42 1879–1887. 10.1002/hbm.25336 33400306 PMC7978128

[B50] Zou Q. H. Zhu C. Z. Yang Y. Zuo X. N. Long X. Y. Cao Q. J. (2008). An improved approach to detection of amplitude of low-frequency fluctuation (Alff) for resting-state fmri: Fractional Alff. *J. Neurosci. Methods* 172 137–141. 10.1016/j.jneumeth.2008.04.012 18501969 PMC3902859

